# Insights into the Role of *Streptomyces hydrogenans* as the Plant Growth Promoter, Photosynthetic Pigment Enhancer and Biocontrol Agent against *Meloidogyne incognita* in *Solanum lycopersicum* Seedlings

**DOI:** 10.3390/plants9091109

**Published:** 2020-08-27

**Authors:** Nandni Sharma, Kanika Khanna, Rajesh Kumari Manhas, Renu Bhardwaj, Puja Ohri, Jawaher Alkahtani, Mona S. Alwahibi, Parvaiz Ahmad

**Affiliations:** 1 Department of Zoology, Guru Nanak Dev University, Amritsar, Punjab 143005, India; sharmanandni1303@gmail.com; 2 Department of Botanical and Environmental Sciences, Guru Nanak Dev University, Amritsar, Punjab 143005, India; kanika.27590@gmail.com (K.K.); renubhardwaj82@gmail.com (R.B.); 3 Department of Microbiology, Guru Nanak Dev University, Amritsar, Punjab 143005, India; rkmanhas@rediffmail.com; 4 Botany and Microbiology Department, College of Science, King Saud University, Riyadh 11451, Saudi Arabia; jsalqahtani@ksu.edu.sa (J.A.); malwahibi@ksu.edu.sa (M.S.A.); 5 Department of Botany, S.P. College Srinagar, Jammu and Kashmir 190001, India

**Keywords:** *Meloidogyne incognita*, *Streptomyces hydrogenans*, photosynthesis, oxidative damage, antioxidants, confocal microscopy

## Abstract

Root-knot nematodes (RKN), *Meloidogyne* sp. hinders functioning of crops and causes global losses in terms of productivity and yield. *Meloidogyne* sp. are microscopic, obligatory endoparasites with ubiquitous distribution in different parts of the world. Taking into consideration these aspects, the present study was conducted to explore nematicidal activity of the *Streptomyces hydrogenans* strain DH-16 against *M. incognita* to regulate its pathogenicity in plants. In-vitro experimentation revealed that pretreated seeds with solvent and culture supernatant lowered root galls in infested plants and promoted growth of *Solanum lycopersicum* seedlings, revealed through the morphological analysis. Additionally, antioxidative defense responses were induced with microbes. However, oxidative stress markers were considerably reduced after microbial inoculations. Apart from this, secondary metabolites were assessed and modulated in RKN infested plants on microbial supplementations. Confocal studies evaluated glutathione accumulation within root apices and its enhancement was directly proportional to defense responses. Therefore, the current study concluded the role of *S. hydrogenans* in stimulating antioxidant potential against RKN along with growth promoting aids. Thus, the outcome of the current study endorses that metabolites produced by *S. hydrogenans* can be used as safe biocontrol agents against *M. incognita* and also as plant growth promoting agents.

## 1. Introduction

Nematodes form a highly diverse group comprising free-living as well as plant and animal parasitic nematodes that can be found worldwide in various habitats [1]. Many species of plant parasitic nematodes (PPNs) can act as pests on a wide range of important agricultural crops. Among PPNs, root knot nematodes (RKNs), are the major destructive pests that infect various agricultural crops globally, especially in tropical and subtropical areas, and cause significant yield losses annually [2]. Plant–parasitic nematodes caused annual yield loss estimated as 8.8–14.6% of the total crop production [3]. In India, the crops susceptible to RKNs include okra [4], tomato [5], cowpea [6], *Withania somnifera* [7], etc. Basically, the *Meloidogyne* spp. are highly destructive due to their rapid multiplication rate and wide host range. *Meloidogyne* spp. have been found to penetrate the epidermal tissue of plant root with the help of several hydrolytic enzymes secreted from the stylet [8,9]. After invasion, the juveniles migrate from root tips to apical meristem where they become established as sedentary root endoparasites [10,11,12,13,14]. This leads to the disruption of root morphology, which also has an impact on root exudation [15]. Generally, root exudates constitute various essential nutrients [16], which gets affected by the infestation of RKNs into the host plants [17]. The visible symptoms of RKNs infected plants include growth impairment, chlorosis and reduction in the level of photosynthetic pigments [18]. Infestation of these nematodes inside the plant tissue causes the oxidative burst that results in the production and generation of reactive oxygen species (ROS), like hydrogen peroxide (H_2_O_2_), superoxide anions, malondialdehyde (MDA), hydroxyl radicals, etc. ROS affects the plant tissue by inducing signalling pathways inside infected plants. These ROS are capable of reacting with membrane proteins, lipids, carbohydrates and thus resulting in the death of cells. In order to neutralise the effect of ROS, various antioxidative enzymes as well as non-enzymatic antioxidants are activated in infected plants. Antioxidative enzymes include catalase (CAT), superoxide dismutase (SOD), ascorbate peroxidise (APOX), polyphenol oxidase (PPO), glutathione-S-transferase (GST), etc. Non-enzymatic antioxidants include total glutathione content, total tocopherol content, flavonoid content, anthocyanin content, etc. [19,20].

*S. lycopersicum*, an important agricultural crop due to its high nutrient content, is grown globally [21]. This crop is highly nutritious and also an important source of essential amino acids, carbohydrates, minerals, various vitamins (A, C and E) and various antioxidants, which are involved in strengthening the defense system of organisms [22]. About 50% of tomato yield losses in India are attributed to PPNs [23,24]. There are various methods that are used for controlling the PPN’s population. Crop rotation is one of the management strategies but is not suitable for controlling RKNs because of the wide distribution of this pathogen. So, another option is the use of chemical nematicides, which are available in the market. These chemicals have been found to work quickly and efficiently, but they also have negative impact on non-target organisms. Thus, side effects of these hazardous chemical nematicides have emphasised the need for new suitable biological methods to control nematodes. It has been found that rhizospheric organisms act as an initial barrier against pathogens attacking plant roots [25]. These agents can act beneficially against RKNs [26,27]. These microbes can act on a variety of nematode hosts that exists in different environmental conditions [28]. Rhizospheric agents include various microorganisms like fungi, bacteria and arbuscular fungi, which are already present in the soil biota [29]. Studies have been reported in which different fungal species like *Lecanicillium muscarium*, *Trichoderma* sp., *Arthrobotrys oligospora*, etc., were used as active nematophagous agents [30,31,32,33]. Arbuscular mycorrhizal fungi like *Scutellospora heterogama*, *Funneliformis mosseae* and *F. versiforme* have also been found as important agents that act as nematode antagonists [34,35,36]. In addition to these, many bacterial species have been reported to act as potential nematode killers. These bacterial species belonging to genera *Bacillus*, *Pseudomonas*, *Burkholderia*, *Streptomyces*, etc., show active responses against RKNs [37,38,39,40,41,42,43,44]. Various *Streptomyces* species including *S. hydrogenans* strain DH-16, *S. antibioticus* strain M7 have been reported to produce active secondary biometabolites that have the capability to act as potential biocontrol agents against *M. incognita*. These biometabolites also have the potential to promote plant growth and also help in water and nutrient uptake and promote plant health [45]. Production of secondary metabolites by microbes having nematicidal properties has also been reported by Borah, et al. [46] and Zhai, et al. [47]. These metabolites have also been found to enhance the defense system and antioxidant potential in plants exposed to RKNs [33,48,49]. All these studies indicate that these biocontrol agents are suitable for the management of RKNs.

The current work was designed to control RKN pathogenesis in *S. lycopersicum* plants using an efficient microbe, the *S. hydrogenans* strain DH16. Presently, a report cites the mortality of J2 (infective stage) of *M. incognita* using the metabolites produced by *S. hydrogenans* strain DH-16 [40]. Thus, carrying forward, the present study was designed to test the biocontrol effects of *S. hydrogenans* strain DH-16 on the RKN disease in tomato and to assess the role of metabolites produced by these microbes in modulating the defense mechanism in 7-day-old *M. incognita* infected *S. lycopersicum* seedlings.

## 2. Results

### 2.1. Morphological Parameters

**Figure 1:**
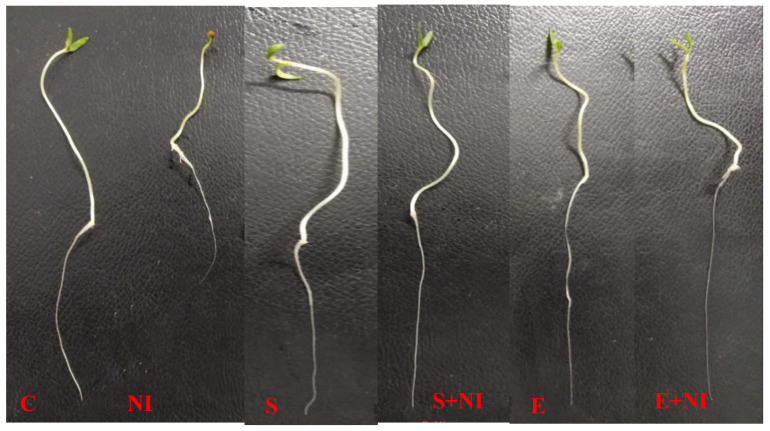
This figure depicts the change in morphometric parameters of tomato seedlings that were pretreated with a supernatant and extract. Different treatments are in order **C** (control), **NI** (nematode inoculated), **S** (supernatant), **S + NI** (supernatant + nematode inoculated), **E** (extract) and **E + NI** (extract + nematode inoculated).

The influence of *S. hydrogenans* strain DH-16 was investigated on various growth parameters like germination %, root length, shoot length, root weight, shoot weight and number of galls (Table 1). All these parameters except the number of galls showed a significant difference in the present study. Nematode infestation decreased the root length, shoot length, root weight and shoot weight by 22%, 5.4%, 13% and 5% respectively, as compared to control seedlings. However, plants that were pretreated with supernatant improved germination %, root length, shoot length, root weight and shoot weight of seedlings by 23%, 3.9%, 24%, 23% and 30.2%, respectively when compared with untreated nematode inoculated seedlings. Moreover, amendment of solvent extract also increased the germination %, root length, shoot length, root weight and shoot weight by 21%, 6%, 3%, 25% and 9% respectively as compared to the seedlings subjected to nematode infection. Supernatant and solvent extract pre-treated seedlings showed a reduced number of galls in roots by 19.9% and 16% respectively, when compared with untreated nematode inoculated seedlings (Table 1, Figure 1).

### 2.2. Effect of Biometabolites Produced by S. hydrogenans Strain DH-16 on the Photosynthetic Pigment of S. lycopersicum Seedlings 7 DAI

**Figure 2:**
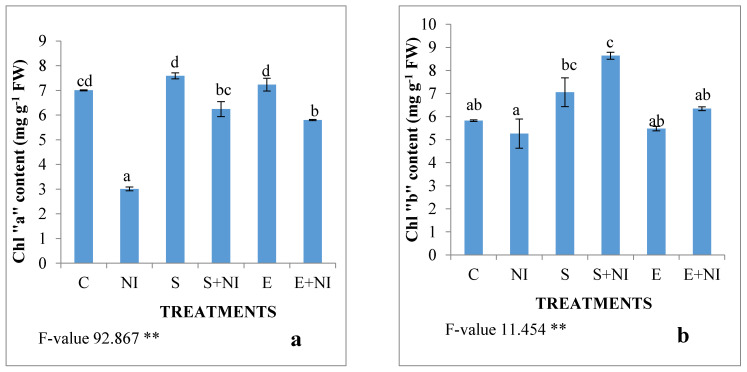

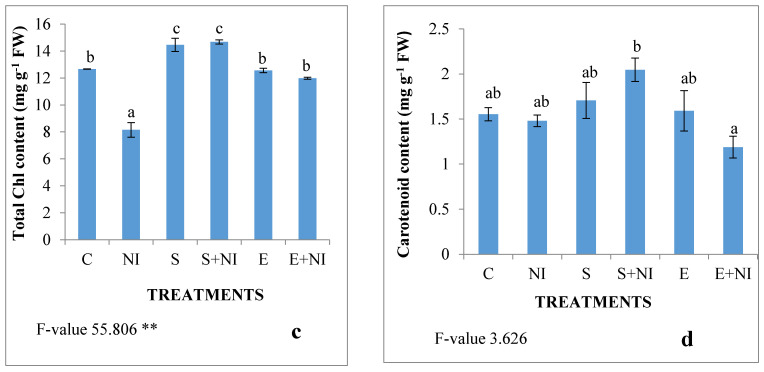
Effect of the supernatant and extract containing biometabolites on the photosynthetic pigments (**a**) Chl “a”, (**b**) Chl “b”, (**c**) Total chl content and (**d**) Total Carotenoid content in 7-day-old *L. esculetum* nematode infected seedlings. Values are presented as means ± standard error (S.E.). F-values ** indicates significance at *p* ≤ 0.01 and * indicates significance at *p* ≤ 0.05. Various alphabets on the graphs indicate that the average mean values of treatments are significantly different according to Tukey’s multiple comparison test (C—Control, NI—Nematode, S—Supernatant, S + NI—Supernatant + Nematode inoculated, E—Extract, E + NI—Extract + Nematode inoculated).

Photosynthetic pigments such as chlorophyll “a”, chlorophyll “b”, total chlorophyll and carotenoid content were assessed (Figure 2a–d). The content of all these pigments was reduced in nematode infested seedlings by 57%, 9.7%, 35.6% and 4% respectively when compared with the control seedlings. The application of the supernatant resulted into increased levels of all the pigments by 107%, 64%, 80% and 37% respectively. The seedlings pre-treated with solvent extract enhanced the levels by 92%, 20%, 47% and 20% respectively when compared with untreated nematode infested seedlings.

### 2.3. Malondialdehyde (MDA) and H_2_O_2_ Content

**Figure 3:**
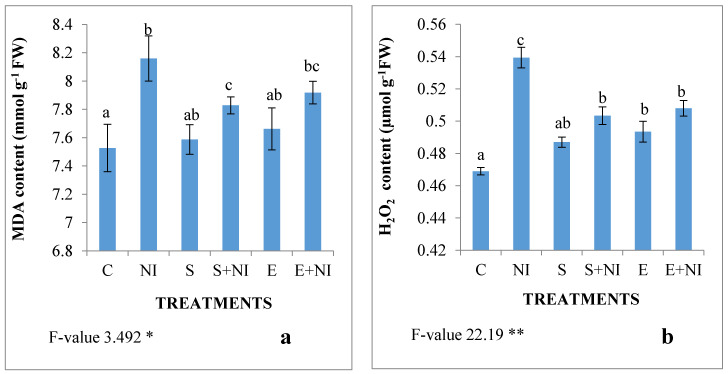
Effect of the supernatant and extract containing biometabolites on the oxidative damage (**a**) MDA and (**b**) H_2_O_2_ content in 7-day-old *S. lycopersicum* nematode infected seedlings. Values are presented as means ± standard error (S.E.). F-values ** indicates significance at *p* ≤ 0.01 and * indicates significance at *p* ≤ 0.05. Various alphabets on the graphs indicate that the average mean values of different treatments are significantly different according to Tukey’s multiple comparison test (C—Control, NI—Nematode inoculated, S—Supernatant, S + NI—Supernatant + Nematode inoculated, E—Extract, E + NI—Extract + Nematode inoculated).

The oxidative damage in nematode infected seedlings was assessed in the form of hydrogen peroxide and MDA content. Nematode infestation resulted in the increased level of MDA by 8% and H_2_O_2_ by 15% (Figure 3a,b). Treatment of the supernatant lowered the contents of MDA by 4% and H_2_O_2_ by 7% respectively in nematode infected seedlings. However, treatment of solvent extract reduced the levels of MDA and H_2_O_2_ by 3% and 7.4% respectively.

### 2.4. Antioxidative Enzyme Activities

Different antioxidative enzymes like CAT, SOD, APOX, GuPOX, PPO and GST were also assessed and it was found that the specific activity of all these enzymes was enhanced in nematode infested *S. lycopersicum* seedlings by 8%, 33%, 37%, 15%, 56.6% and 40% respectively in comparison to control seedlings, whereas supernatant pretreated nematode infected seedlings showed further increase in the specific activity of all these antioxidative enzymes by 3%, 15%, 4%, 1%, 15% and 20% respectively when compared with nematode infested untreated seedlings.

Supplementation of solvent extract in nematode infested seedlings resulted in enhanced specific activities of CAT (1.9%), APOX (by 2%), PPO (by 1%), GPOX (by 14%) and GST (by 13.6%) when compared with nematode infected *S. lycopersicum* seedlings (Table 2).

### 2.5. Non-Enzymatic Antioxidants

**Figure 4:**
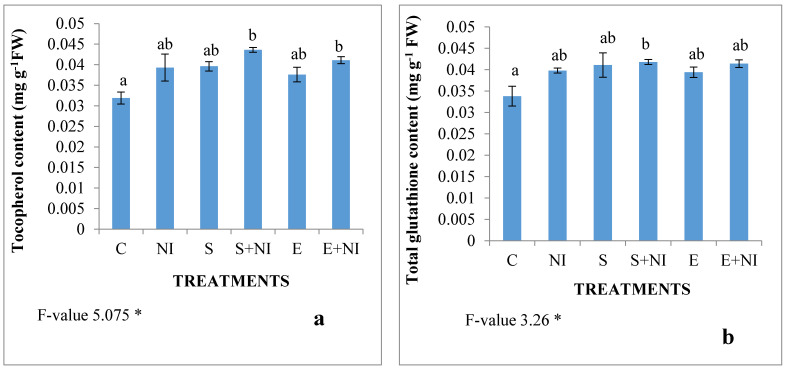
Effect of the supernatant and extract containing biometabolites on the non-enzymatic antioxidants (**a**) Tocopherol and (**b**) Glutathione content) in 7-day-old *S. lycopersicum* nematode infected seedlings. Values are presented as means ± standard error (S.E.). F-values ** indicates significance at *p* ≤ 0.01 and * indicates significance at *p* < 0.05. Various alphabets on the graphs indicate that the average mean values of different treatments are significantly different according to the Tukey’s multiple comparison test (C—Control, NI—Nematode inoculated, S—Supernatant, S + NI—Supernatant + Nematode inoculated, E—Extract, E + NI—Extract + Nematode inoculated).

The levels of non-enzymatic antioxidants like glutathione and tocopherol content were assessed and were found to play an important function in nematode infested plants and the inoculation of biometabolites produced by microbes boosted their activities. The level of tocopherol and glutathione was enhanced by 23% and 18% respectively in nematode infected seedlings. These contents further increased in nematode infested seedlings that were pretreated with supernatant by 11% and 5% respectively, when compared with untreated nematode infected seedlings. However, the level of tocopherol and glutathione was enhanced in the nematode infected solvent extract treated seedlings by 12% and 4% respectively in comparison to the nematode contaminated *S. lycopersicum* seedlings (Figure 4a,b).

### 2.6. Phenolic Compounds

**Figure 5:**
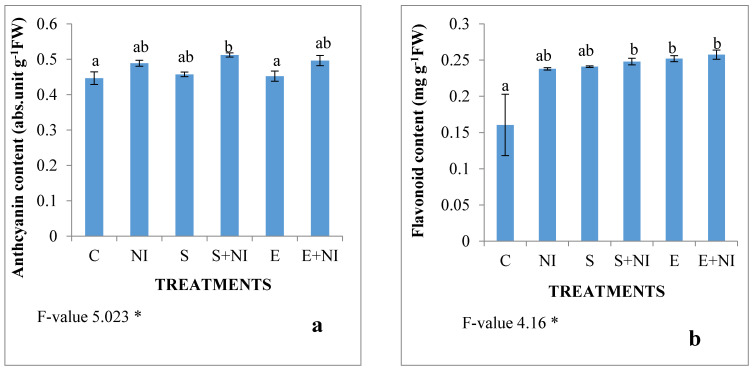
Effect of the supernatant and extract containing biometabolites on the phenolic compounds: (**a**) anthocyanin and (**b**) flavonoid content in 7-day-old *S. lycopersicum* nematode infected seedlings. Values are presented as means ± standard error (S.E.). F-values ** indicates significance at *p* ≤ 0.01 and * indicates significance at *p* < 0.05. Various alphabets on the graphs indicate that the average mean values of different treatments are significantly different at *p* < 0.5 according to the Tukey’s multiple comparison test (C—Control, NI—Nematode inoculated, S—Supernatant, S + NI—Supernatant + Nematode inoculated, E—Extract, E + NI—Extract + Nematode inoculated).

Effect of *S. hydrogenans* on phenolic compounds such as the anthocyanin content, flavonoid content was also studied in the present work. The levels of anthocyanins and flavonoids increased in the *M. incognita* infected seedlings by 9% and 48% respectively. Furthermore, in the supernatant treated nematode infested seedlings, the level of anthocyanin and flavonoid content increased by 5% and 4% respectively. Whereas, in the seedlings pretreated with solvent extract, the level of anthocyanin and flavonoid content was enhanced by 2% and 10% respectively, when compared with the untreated nematode inoculated seedlings (Figure 5a,b).

### 2.7. Confocal Microscopy

**Figure 6:**
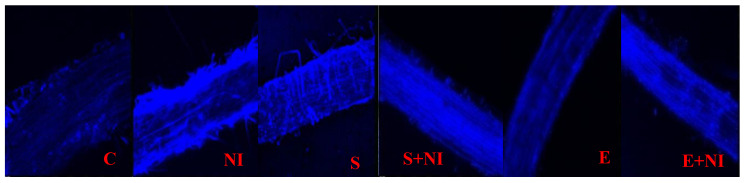
Confocal micrographs showing images of glutathione tagging in differently treated control (**C**), nematode inoculates (**NI**), supernatant (**S**), supernatant (**S + NI**), extract (**E**) and extract + nematode inoculated (**E + NI**) *S. lycopersicum* roots 7 DAI by staining them with MCB.

Tagging of glutathione was performed by dipping tips of roots of *S. lycopersicum* seedlings in MCB dye. Tagging was noticed on the basis of intensity of the blue colour in comparison to control seedlings. It was found that the root tissue of nematode infected seedlings revealed high intensity of blue colour, i.e., high level of glutathione content when compared to untreated control seedlings. Moreover, this intensity was further increased in the seedlings pretreated with biometabolites present in culture filtrate and extract, indicating the antioxidative features of biometabolites produced by *S. hydrogenans* strain DH-16 (Figure 6).

## 3. Discussion

The present study revealed a remarkable effect of biometabolites produced by *S. hydrogenans* strain DH-16 in mitigating nematode stress in *S. lycopersicum* seedlings. *M. incognita* had a negative impact on the morphological parameters of infected *S. lycopersicum* seedlings. In the presence of *M. incognita*, there was an overall decline in the length and weight of *S. lycopersicum* seedlings. Our studies are in agreement to the previous studies in which reduction in morphological attributes was depicted in *Medicago truncatula* plants. They observed an enhanced formation of galls over root surfaces due to nematode infestation [50], which in turn impaired the vascular transportation in plants. In addition, Venkatesan, et al. [51] observed a hindrance in the mineral uptake of *Oryza sativa* plants due to nematode infections. They concluded that nematodes clogged the vascular pathways of plants by their rapid multiplication within the tissues leading to overall impairment in their metabolic activities [51]. A similar decline in the fresh weight was observed in *Ocimum basilicum* plants infected with *M. incognita* [52]. According to them, nematode infestation had a negative impact on the uptake of nutrients by plants. The plants overcome such harsh situations via developing the lateral root elongation in order to increase the nutrient uptake from the soils [53]. This characteristic feature not only balances the nutritional homeostasis, but also restricts the nematodes towards outer zones [54]. The retardation of growth parameters in the present study could be due to the reason of root damage, which act as a hurdle in mineral absorption from the root system and their translocation towards the shoot, thus disrupting the complete physiology of plant. Moreover, earlier studies carried out by Gupta, et al. [7] in *Withania somnifera* also demonstrated similar results where morphological traits were reduced in terms of height as well as biomass. Our study also coincides with Khajuria and Ohri [55], Khanna, et al. [56] and Khanna, et al. [57] in tomato plants in terms of decreased root length, shoot length, fresh weight and dry weight on nematode infestations. They inferred that the blockage of pathways associated with nutrition and water transport via nematodes leads to retarded growth attributes [56,57].

Treatment with biometabolites produced by *S. hydrogenans* strain DH-16 in the form of the supernatant and extract resulted in a decreased number of galls, and stimulated root length, shoot length and fresh and dry weights of *M. incognita* infected *S. lycopersicum* seedlings. Similar results were found in cucumber plants pretreated with the culture broth of *Streptomyces* isolates NA-303 [58]. They found a remarkable suppression in the number of galls in microbe treated seedlings, directly influencing growth and development of plants. According to them, nematode motility causing substances were produced by *Streptomyces* isolates respectively. Our results were also in agreement with Khanna, et al. [56] and Khanna, et al. [57] who revealed that inoculations of *Pseudomonas aeruginosa* and *Burkholderia gladioli* promoted growth parameters in *M. incognita* infected *S. lycopersicum* plants and also reduced gall formation. They speculated that microbes restrained the nematode entry within the plant cells by forming a uniform layer over root surfaces to avoid their entry within the plants [56,57]. The most possible mechanism behind microbial mediated nematicidal activity in the present study could be due to production of toxic nematicidal compounds by these microorganisms, which caused nematode mortality and ultimately resulted in the plant growth promotion.

The results obtained from present study also determined a decline in the content of photosynthetic pigments in *M. incognita* infected *S. lycopersicum* seedlings. The similar reduction in the levels of pigments like chlorophyll, carotenoid and β-carotene was revealed earlier by Vasil’Eva, et al. [59]. This decline in plant pigments is mainly because of the inhibition of crucial enzymes required in the Violoxanthin pathway, this in turn impairs the stability of the photosynthetic apparatus [59]. Likewise, Sharma, et al. [18] reported lowered or disrupted photosynthetic pigments in tomato plants subjected to nematode infections. Reduction in the plant pigments are attributed to the formation of galls over root surfaces, leading to disrupted water transportation and chlorophyll a and b levels [60]. Our study also revealed inclined levels of photosynthetic pigments in *S. hydrogenans* treated *S. lycoperscum* seedlings infested with *M. incognita*. Our findings are supported from the previous studies conducted by Sharma, et al. [18] in tomato plants. They observed that plants treated with *S. antibioticus* strain M7 showed an enhancement in the levels of pigments due to triggered photosynthetic activities of plants. Moreover, a study reported by Jiang, et al. [61], also revealed that *Trichosanthes kirilowii* plants infested with nematodes showed an increase in pigment levels upon microbial inoculations. Another study reported by Khanna, et al. [57] suggested that stimulatory effects in their photosynthetic apparatus are due to microbe mediated upregulation in the enzymes responsible for pigment synthesis. Apart from this, they also concluded that the role of microbes in plant growth promotion in terms of nitrogen fixation, siderophore synthesis, etc., also leads to their better metabolic functions [57]. Furthermore, da Silva, et al. [62] observed better photosynthesis in nematode inhabited *Pinus pinaster* under the treatment of microbial strains. Present study upholds the fact that treated plants show better functions due to their ability to suppress nematode functioning or might be due to its ability to enhance enzymes associated with pigment production during nematode infection.

Our work also revealed an enhancement in the oxidative damage in nematode infected *S. lycopersicum* seedlings in the form of increased oxidative stress markers in terms of MDA and H_2_O_2_ content in *M. incognita* stressed plants. A similar increase in the level of MDA was also reported in case of sugar beet plants inoculated with nematodes [63]. Likewise, enhancement in the H_2_O_2_ content was also assessed by Sharma and Sharma [64] in tomato plants. They suggested that nematode infestation led to the generation of ROS in host plants. It is attributable to the pathogenicity of nematodes in the form of elevated ROS within stressed plants. Moreover, a tremendous increase in ROS species in the *Bacopa monnieri* plants has also been reported upon nematode infestations [39]. Along with this, studies conducted by Khanna, et al. [56] also formulated a paramount increase in oxidative stress markers in tomato plants infested with nematodes. However, the present work also assessed the role of microbes on oxidative damage in plants inhabited with nematodes. It was observed that the seedlings pretreated with biometabolites reduced the level of oxidative stress markers. Our findings were in agreement with Khanna, et al. [56] in *S. lycopersicum* plants treated with *P. aeruginosa* and *B. gladioli* who revealed declined levels of MDA and lipid peroxidation in plants. According to their study, this reduction is due to enhanced activities of defense enzymes in microbe inoculated plants [56]. Similarly, Gupta, et al. [39] conducted research in nematode infected *B. monnieri* plants inoculated with *Streptomyces* sp. and *Chitinphilus* sp. in which H_2_O_2_ and MDA content was found to be considerably reduced. They speculated that this decrease is mainly due to the production of immunity developed signals via microbes in order to combat pathogen accumulated within plants [39]. Furthermore, microbes possess the ability to scavenge the ROS produced by plants through the ability to secrete chelating molecules like organic acids, siderophores, and other secondary metabolites. Moreover, plants have the inbuilt capability to protect themselves from different stresses by inducing the activities of the antioxidative defense system in terms of enzymatic and non-enzymatic antioxidants [65]. The present study revealed an increase in the activities of various enzymes (CAT, SOD, APOX, GPOX, PPO and GST) in *M. incognita* infected *S. lycopersicum* seedlings. Similar results were also reported by Khajuria and Ohri [55] in the case of tomato plants, where activities of SOD, POD, CAT, GPOX, etc., were enhanced during nematode infection. As per the reports of Alscher, et al. [66] the activities of defensive enzymes occurred in response to increased oxidative stress markers in plants. In addition to this, enhancement in the activities of SOD, POD and CAT was also found in tomato plants under nematode influence [67]. This mainly relied on the basis of modulation in the defensive pathways. Our study also demonstrated a further incline in the enzymatic activities after the treatment with the biometabolites produced by *S. hydrogenans* in the form of the supernatant and extract. A rise in the activity of PPO was also reported by Ma, et al. [68] in *Streptomyces* sp. treated nematode infected tomato plants. According to their speculation, this inflation in enzymatic activity is correlated to tolerance towards the pathogenic response via systematic resistance [68]. Additionally, the results of Gupta, et al. [7] reported an increase in the activity of SOD in *Withania somnifera* plants, treated with *Streptomyces* sp. and *Chitiniphilus* sp. respectively, due to the internal immune response activation along with induced systemic resistance. The activities of CAT, SOD, PPO and GuPOX were also triggered in nematode infected tomato plants treated with *P. aeruginosa* and *B. gladioli* as revealed by Khanna, et al. [56]. The reason behind this remarkable increase in antioxidative enzyme activities might be due to the increased protein content in plants after supplementation of beneficial microbes, which further resulted in upregulation of these defense enzymes in the host plants.

In addition to antioxidative enzymes, non-enzymatic antioxidants like glutathione content and tocopherol content were also found to be enhanced in nematode infected *S. lycopersicum* seedlings. As depicted in the previous reports, glutathione content acts actively in reducing the contents of thiol-sulfide and sulphur present inside the cells and has also found to help the plants in mitigating abiotic and biotic stresses [69]. Tocopherol, mainly present in the membranes of chloroplast, acts as a lipid peroxyl scavenger in most of the plants [70]. The levels of these non-enzymatic antioxidants were enhanced in infected plants like tomato, eggplant, pea, papaya and grapevine under the incidence of nematode infection [56,57,70,71]. Moreover, an elevation in the levels of tocopherol was also observed in *Helianthus annuus* plants subjected to nematode infestation [72]. They found this enhancement in close linkage with the defense potential of plants against inhabiting nematodes [72]. Further, the glutathione and tocopherol levels were found to be enhanced in seedlings treated with biometabolites produced by *S. hydrogenans*. A study conducted by Khanna, et al. [56] also revealed similar trends in which nematode infected tomato seedlings demonstrated raised levels of non-enzymatic antioxidants after inoculation with *P. aeruginosa* and *B. gladioli* respectively. According to their perception, these increased levels might be due to the activation of antioxidants by soil microbes and in order to act as scavengers by reducing H_2_O_2_ to H_2_O [73,74]. This further reduces the oxidative stress from the cells through the growth promoting action of microbes in complicated situations. Moreover, the induction in the level of antioxidants in nematode stressed plants in the present study might be due to the fact that microbes boosted the internal immune system within the plants to form a shield against the adversities caused by RKNs.

The current study also determined an elevation in the levels of phenolic compounds like flavonoids and anthocyanins in *M. incognita* infected *S. lycopersicum* seedlings. It has been elucidated since previous times that phenols act as buffers to regulate the stress levels in plants [75]. Moreover, many different categories of phenolic compounds act as nematicidal elements to cope up with the RKN infections [76]. Along with this, they also possess the characteristics to subdue the gall formation in plants and maintain the proper regulatory actions of the plants [77]. Induction in the levels of phenols have been directly in proportion to the stress levels as it gets deposited onto the cell wall in order to toughen them against a pathogen attack [77]. Various studies revealed that enhancement in the levels of different phenolic compounds in tomato plants against nematicidal attack to counteract the responses [78]. Our study also found support by the findings of Treutter [79], who confirmed that this rise is associated with the phytopathogen infection. Apart from this, the enhanced level of phenolic compounds also reduces the infestation of pathogens within plants [5,80]. Our results also revealed that the level of these compounds was further increased in the seedlings pretreated with biometabolites produced by *S. hydrogenans*. This enhancement or accumulation might be linked with the biotic stress tolerance as indicated by a reduction in the nematode infestation by impairing gall formation. Our results were also on similar lines as with Gupta, et al. [7], in the case of nematode infected *W. somnifera* treated with *Chitiniphilus* sp., *Streptomyces* sp. and *Cellulosimicrobium* sp. respectively. They depicted that this increase in the phenolic compounds led to normal functioning of plants during a pathogenic attack. Additionally, inclined levels of phenolic compounds, flavonoids, anthocyanins and polyphenols were also found in nematode infested tomato plants under the incidence of a microbial treatment [57]. They also depicted an upregulation in the activities of genes encoding phenolic compounds that is *PAL* and *CS* during microbial treatment in infected plants. Their studies recapitulated that phenolic compounds triggered the immune responses within plants in order to combat the mechanical injuries caused to them by pathogens along with mediating normal metabolic activities of the plants [57]. The induction in the level of phenolic compounds such as anthocyanins and flavonoids in the present study might be due to the chelating action of these compounds against pathogenic responses. Moreover, the synthesis of these compounds also hinders the movement of nematodes, thereby, directly targeting them to cause their morbidity. All these observations indicated the efficiency of biometabolites produced by *Streptomyces* sp. In developing stress tolerance in *M. incognita* infected host plants, thereby, making them the perfect candidates as biocontrol agents against nematode infections.

## 4. Materials and Methods

Nematode culture: Culture of nematodes was collected from infected roots of tomato and brinjal from different sites of Guru Nanak Dev University campus. Females were isolated and identified. The identified culture was then used for maintaining the pure cultures in the glass house of the department and then used for further studies. Egg masses of *M. incognita* were extracted from the pure culture, washed with 1.5% sodium hypochlorite solution [81], and then subsequently washed with distilled water. These egg masses were then kept at temperature 26 ± 2 °C in a Biological Oxygen Demand (B.O.D) incubator (CALTAN, NSW, New Delhi, India) to stimulate hatching. After 2–3 days, second stage juveniles (J2) were collected and were counted using alight microscope and used for further experimental work.

Microbial inoculations: *S. hydrogenans* strain DH-16 (Gene Bank accession no. JX123130) was obtained from the Department of Microbiology, Guru Nanak Dev University, Amritsar (Punjab) [82]. The bacterial strain was prepared on starch casein nitrate agar (SCNA) plates at a temperature of 4 °C and was then stored at 4 °C for further studies.

### 4.1. Production of Secondary Metabolites

The bioactive metabolites production by *S. hydrogenans* strain DH-16 was carried out as described by Kaur and Manhas [82]. The production of metabolites was carried out in the Orbital shaker incubator at 28 °C temperature and 180 rpm. After incubation of 72 h under the required conditions, the culture broth was collected and centrifuged at 10,000 rpm at 4 °C for 20 min. The filterate/supernatant (free from cells) were used for experimental studies.

The bioactive metabolites in the filtrate were extracted using ethyl acetate and were concentrated using rota evaporator (IKA India Pvt. Ltd., RV 10 digital V, Bengaluru, India). The resulted brown coloured compound thus produced was redissolved in 0.5% DMSO and stored at 4°C for further experimental work.

### 4.2. Plant Material and Treatments

Surface sterilized *S. lycopersicum* seeds var. Pusa Ruby (susceptible variety) were soaked for 5 h in the supernatant (cell free filtrate) as well as in the crude extract (480 µg/mL). The seeds were grown in autoclaved petri plates lined with whatman filter paper grade 1. Each petri plate contained 25 seeds with treatment of 0.5% DMSO solution (control) and the crude extract (E) as well as the cell free supernatant (S). After germination, the seedlings were inoculated with 5 juveniles per seedling in all the treatments. The petri plates were kept in B.O.D incubator “CALTAN” (Super Deluxe Automatic) for 7 days with a light period of 16 h per day. After 7 days of nematode inoculation, the experiment was terminated and various morphological and physiological parameters were studied. For each set of experiment, treated and inoculated plants were compared with untreated, uninoculated plants as well as untreated, inoculated plants.

### 4.3. Morphological Parameters

After 7 days of nematode inoculation, various morphological parameters were studied. The parameters include percentage germination, root length, shoot length, root weight, shoot weight and number of galls.

### 4.4. Determination of Carotenoid and Chlorophyll Contents

Extraction of carotenoid and chlorophyll content was done using 80% acetone [83,84]. Fresh leaf tissue of seedlings was crushed in 80% acetone in the 1:4 ratio and then subjected to centrifugation at 4 °C at 10,000 rpm for 20 min. The supernatant was removed and its absorbance was recorded at 480 and 510 nm for carotenoid content and 645 and 663 nm for chlorophyll content using a UV-VIS spectrophotometer (GENESYS 108, Waltham, MA, USA).

### 4.5. Determination of Malondialdehyde (MDA) Content and H_2_O_2_ Content

MDA content was used for the measurement of lipid peroxidation [85]. Homogenate was prepared by crushing fresh plant seedling in 0.1% (w/v) trichloroacetic acid (TCA), followed by centrifugation at 4 °C at 5000 rpm. The supernatant thus produced was used for further analysis. Of the supernatant 1 mL was mixed with 6 mL of 20% (w/v) TCA containing 0.5% (w/v) TBA and was heated at 95 °C for 30 min in a water bath and then the reaction was terminated in ice cold conditions. The optical density of the supernatant was taken at 532 nm. The nonspecific absorbance was corrected by subtracting absorbance at 600 nm. The level of MDA content was determined by using 155 mM cm^−1^ as an extinction coefficient.

For H_2_O_2_ content, the homogenate was prepared by crushing 0.5 g whole seedling in 1% trichloroacetic acid (TCA) and centrifuged at 12,000 rcf at 4 °C for 15 min. For further analysis, 0.5 mL of supernatant was mixed with 0.5 mL potassium phosphate buffer of pH 7 (PPB) and 1 mL potassium iodide (KI, 1 M). The absorbance of the mixture was then measured at 390 nm. A standard curve of H_2_O_2_ was used for evaluation [86].

### 4.6. Protein Content and Antioxidative Enzymes

Sample preparation: Fresh *S. lycopersicum* seedlings were homogenised in 0.1 M potassium phosphate buffer having pH 7.0. Homogenate was then subjected to centrifugation at 13,000 rpm at 4 °C for 20 min. The protein content was estimated by using the Folin Ciocalteu phenol reagent [87] and bovine serum albumen (BSA) was used as a standard for the evaluation of protein content. The activities of various antioxidative enzymes were determined by standard protocols reported by Aebi [88] for catalase (CAT; E.C. 1.11.16), Kono [89] for superoxide dismutase (SOD; E.C. 1.15.1.1), Nakano and Asada [90] for ascorbate peroxidase (APOX; E.C. 1.11.11.1), Esterbauer, et al. [91] for polyphenol oxidase (PPO; E.C. 1.10.3.1), Pütter [92] for guaiacol peroxidise (GuPOX; E.C. 1.11.1.7) and Habig and Jakoby [93] for glutathione-S-transferase (GST; E.C. 2.5.1.18).

### 4.7. Non Enzymatic Antioxidants

Total glutathione content: Total glutathione content was determined using the method given by Sedlak and Lindsay [94]. The homogenate was prepared by crushing whole seedling in 50 mM Tris buffer (pH 10), followed by centrifugation at 12,000× *g* at 4 °C for 15 min, the supernatant thus formed was mixed with DTNB and methanol and was kept at room temperature for 15 min. Incubation was followed by centrifugation at 3000× *g* for 15 min and the absorbance of the supernatant was measured at 412 nm. A standard plot was generated using the known concentration of reduced glutathione for evaluation.

Tocopherol content: The homogenate was prepared in a similar way as in the case of the total glutathione content. The supernatant was mixed with absolute ethanol and double distilled water, and the mixture thus formed was shaken. Then xylene was added, followed by centrifugation at 3000× *g* at 4 °C for 10 min. Then the upper xylene layer was separated and was mixed with 2,4,6-tripyridyl-s-triazine and absorbance was measured at 600 nm. A standard plot was generated using the known concentration of tocopherol for evaluation [95].

### 4.8. Estimation of Phenolic Compounds

Flavonoid content: The homogenate was prepared by crushing fresh seedlings in 80% methanol, followed by centrifugation at 4 °C and at 10,000 rpm for 20 min. The supernatant thus formed was used for estimation of flavonoid content. The 100 µL of the supernatant was taken in a test tube and then with the addition of methanol final volume of 3 mL was made. To this mixture 100 µL of AlCl_3_·6H_2_O, 100 µL of 5% sodium potassium tartarate and 0.5 mL of distilled water were added sequentially. The final mixture thus formed was shaken vigorously and was then the absorbance was recorded at 415 nm after 30 min of incubation. A standard plot was generated using known concentration of rutin. [96].

Anthocyanin content: The anthocyanin levels were estimated by crushing fresh *S. lycopersicum* seedlings in acidified methanol that had been prepared by mixing methanol, distilled water and hydrochloric acid at a ratio of 79:20:1. The extract was kept overnight at 4 °C and was then centrifuged at 10,000× *g* for 15–20 min at 4 °C. The absorbance was read at 530 and 657 nm [97].

### 4.9. Confocal Microscopy

Confocal microscopy was used for studying glutathione content. For glutathione tagging, root tips of *S. lycopersicum* seedlings were dipped in 25 mM monochlorobimane (MCB) solution and were kept in dark conditions for 20 min and extra stain was then washed with deionized water [98]. The samples were further examined by a Nikon A1R confocal laser scanning microscope (CLSM, New York, USA) at an excitation wavelength of 351–364 nm and emission wavelength of 477 nm.

### 4.10. Statictical Analysis

All the calculations (one-way ANOVA and Tukey’s test) were performed by using SPSS software (IBM SPSS, Version 24.0, IBM Corp., Armonk, NY, USA) at *p* ≤ 0.01 and *p* ≤ 0.05 and by self-coded Microsoft office excel software.

## 5. Conclusions

The current study showed alterations in the morphological and physiological characteristics of *M. incognita* infected *S. lycopersicum* seedlings. *S. hydrogenans* strain DH-16 release substantial metabolites had nematicidal activity that also led to activation of defense parameters in the RKN infected host plants. Basically, metabolites produced by these microbes enhanced the stress tolerance level by modulating the defense responses. Moreover, our results also showed an increase in the growth parameters, photosynthetic pigments, phenolic compounds of the nematode infested plants treated with biometabolites in the form of the cell filterate (supernatant) and extract. Furthermore, these metabolites are involved in regulating the level of ROS under stress by elevating the activities of enzymatic and non-enzymatic antioxidants. As a result, the present study suggests that *Streptomyces* sp. play a vital role in biotic stress mitigation in *S. lycopersicum* seedlings. Thus, the biometabolites produced by *S. hydrogenans* strain DH-16 can be used as a potential agent for the management of *M. incognita* along with the benefit of increasing germination as well as growth parameters of infected plants. Moreover, further research is required to elucidate enhanced tolerance of host plants against nematodes by microbial strains.

## Figures and Tables

**Table 1:** Morphological parameters of *S. lycopersicum* 7 days after nematode inoculations (DAI) under different treatments of biometabolites produced by *S. hydrogenans* in the form of a supernatant and solvent extract. Different treatments are represented in tables as C (control), NI (nematode inoculated), S (supernatant), S + NI (supernatant + nematode inoculated), E (extract) and E + NI (extract + nematode inoculated).

Treatments	Parameters (Mean ± S.E.)					
	Germination %	Root Length (cm)	Shoot Length (cm)	Root Weight (g)	Shoot Weight (g)	No. of Galls
**C**	70.67 ± 1.453 ^a^	8.827 ± 0.361 ^bcd^	7.337 ± 0.130 ^a^	0.150 ± 0.012 ^ab^	0.5600 ± 0.021 ^a^	-
**NI**	69.33 ± 1.202 ^a^	6.897 ± 0.529 ^a^	6.943 ± 0.135 ^a^	0.127 ± 0.008 ^a^	0.533 ± 0.015 ^a^	8 ± 0.333 ^a^
**S**	87 ± 1.527 ^b^	9.130 ± 0.460 ^cd^	7.9167 ± 0.202 ^ab^	0.170 ± 0.006 ^b^	0.693 ± 0.009 ^b^	-
**S + NI**	85.67 ± 1.202 ^b^	7.173 ± 0.061 ^ab^	8.620 ± 0.399 ^b^	0.1633 ± 0.003 ^ab^	0.690 ± 0.023 ^b^	6 ± 1.202 ^a^
**E**	86.67 ± 1.333 ^b^	9.150 ± 0.105 ^d^	7.456 ± 0.059 ^a^	0.170 ± 0.100 ^b^	0.690 ± 0.006 ^b^	-
**E + NI**	84 ± 1.732 ^b^	7.287 ± 0.525 ^abc^	7.157 ± 0.206 ^a^	0.153 ± 0.008 ^ab^	0.580 ± 0.012 ^a^	7 ± 0.575 ^a^
**F-value**	33.747 **	7.440 **	7.903 **	3.733 *	23.431 **	52.235 (ns)

The results are presented in the form of mean ± standard error (S.E.) and F-value ** indicates significance at *p* ≤ 0.01, * indicates *p* ≤ 0.05 and ns indicates non-significant difference according to Tukey’s comparison test. Different letters a, b, c and d on table represents significant difference whereas the same letters represent no significant difference.

**Table 2:** Effect of biometabolites produced by *S. hydrogenans* strain DH-16 in the form of supernatant and solvent extract on *S. lycopersicum* seedlings 7 days after nematode inoculation. Different treatments are represented in tables as C (control), NI inoculated (nematode inoculated), S (supernatant), S + NI (supernatant + nematode inoculated), E (extract) and E + NI (extract + nematode inoculated).

Treatments	Parameters (Mean ± S.E.)					
	Catalase (CAT) (U/mg Protein)	Superoxide Dismutase (SOD) (U/mg Protein)	Ascorbate Peroxidise (APOX) (U/mg Protein)	Polyphenol Oxidise (PPO) (U/mg Protein)	Guaiacol Peroxidise (GuPOX) (U/mg Protein)	Glutathione-S-Transferase (GST) (U/mg Protein)
**C**	0.1249 ± 0.0195 ^a^	0.0030 ± 0.00028 ^a^	0.0651 ± 0.00451 ^a^	0.0119 ± 0.00040 ^a^	0.0534 ± 0.00266 ^a^	0.0471 ± 0.00375 ^a^
**NI**	0.1353 ± 0.0175 ^a^	0.0039 ± 0.00028 ^abc^	0.0886 ± 0.00426 ^b^	0.0138 ± 0.00048 ^a^	0.0827 ± 0.00632 ^b^	0.0659 ± 0.01176 ^ab^
**S**	0.1487 ± 0.0106 ^a^	0.0043 ± 0.00017 ^bc^	0.0809 ± 0.00330 ^ab^	0.0130 ± 0.00024 ^a^	0.0715 ± 0.00576 ^ab^	0.0591 ± 0.00452 ^ab^
**S + NI**	0.1407 ± 0.0133 ^a^	0.0045 ± 0.00032 ^c^	0.0918 ± 0.00070 ^b^	0.0140 ± 0.00033 ^a^	0.0954 ± 0.00796 ^b^	0.0791 ± 0.00279 ^b^
**E**	0.1357 ± 0.0042 ^a^	0.0032 ± 0.00010 ^ab^	0.0777 ± 0.00509 ^ab^	0.0128 ± 0.00083 ^a^	0.699 ± 0.00829 ^ab^	0.0503 ± 0.00321 ^ab^
**E + NI**	0.138 ± 0.00493 ^a^	0.0037 ± 0.00015 ^abc^	0.0908 ± 0.00474 ^b^	0.0139 ± 0.00016 ^a^	0.0950 ± 0.00111 ^b^	0.0753 ± 0.00890 ^ab^
**F-value**	0.355 (ns)	6.632 **	6.372 **	3.174 (ns)	7.452 **	3.761 *

The results are presented in the form of mean ± standard error (S.E.) and F-value ** indicates significance at *p* ≤ 0.01, * indicates *p* ≤ 0.05 and ns indicates non–significant difference according to Tukey’s comparison test. Different letters a, b and c on table represents significant difference whereas the same letters represent no significant difference.
